# Luxation of Metatarsal Bones under the Tarsus

**Published:** 1854-11

**Authors:** 

**Affiliations:** Surgeon to the City of Dublin Hospital


### A CASE OF LUXATION OF THE METATARSAL BONES UNDER THE TARSUS —A FORM OF DISLOCATION NOT HITHERTO DESCRIBED.

BY MR. TUFFNELL,

Surgeon to the City of Dublin Hospital.

(*Dublin Quarterly Journal of Medicine*, February, 1854.)

Instances of luxuation of the metarsus *upon* the tarsus are very rare, only six having as yet been recorded, but laxation of the metatarsus *under* the tarsus is still rarer, and the subjoined case is the only one on record.

With regard to the diagnostic sign it will be seen that the foot is shortened three-fourths of an inch or more, curved inwards, and at the base of the great toe broader than its fellow by an inch; that the instep stands out sharply defined, with a sudden angular prominence and marked deficiency in front; that the arch of the foot on its inner border is preserved, but the centre of the sole is occupied by the tarsal extremities of the displaced metatarsal bones. Mr. Tuffnell's account of the case is as follows:

"For the opportunity of witnessing it I am indebted to Mr. Dolmage, surgeon of the 7th Dragoon Guards, in whose regiment the accident occurred, and in the following manner: a trooper was returning off duty to Portobello Barracks, Dublin, on the 30th of November, 1851, and was walking his horse cautiously, the road being very slippery from frost. Whilst turning a corner, bordering upon the canal, the animal suddenly slipped, and fell with his whole weight upon the soldier's right leg and foot, crushing it against the ground. The horse rose instantly, the man remaining in the saddle, but suffering such agony, that, unconscious of what he was doing, he reined the animal back into the canal. Here a violent struggle ensued, the horse eventually disengaging himself from his rider, who, assistance being at hand, was dragged out, and taken to his regimental hospital close by. He was seen by Mr. Dolmage within a very few minutes of the accident having occurred, and before any considerable degree of swelling had taken place.

"The foot was found to be much shortened, curved inwards and bent, the tarsus presenting a hard bony projection, overhanging the metatarsus, whilst deep under tbe plantar structures a second bony mass could be felt lying obliquely across the sole of the foot.

"Reduction was at once attempted by placing the patient on his back, fixing the pelvis, flexing the leg upon the thigh, and extension then made by pulleys attached to the extremity of the foot and to the toes, and persevered in for a considerable time, during which every possible movement of the metatarsus upon the tarsus, calculated to assist reduction, was resorted to, and leverage also made upon the dislocated extremity of the metatarsal bone of the great toe, were projecting in the sole, by means of a ruler being applied to it, and drawn upwards and forwards, whilst the clasped hand of a powerful assistant, placed upon the instep, held that part downwards and backwards. As great a degree of force as it was considered justifiable to employ was expended in the effort at reduction, and continued for one hour, but not the slightest alteration in the position of the bones could be effected. Considerable effusion and ecchymois followed, the latter extending up almost to the knee.—Leeches, fomentations, &c., were prescribed, and the ordinary treatment for violent contusions had recourse to. Under this treatment swelling subsided.

"All swelling and thickening had now disappeared, the outline of the tendons and every portion of the extremity being most accurately defined. In its general aspect, the foot somewhat resembled a case of pes equinus, being considerably shortened and arched upon its inner border, the distal extremity of the metatarsal bone and first phalanx of the great toe being adducted, the last phalanx at the same time pointing somewhat outwards. The instep presented a normal condition from the malleoli to the extremity of the internal cuneiform bone, which projected in a sharp point, raising the integument, which was stretched over it, white and glistening like a tightly bent knuckle; from the outer border of the cuneiform bone ran an evident ridge, marking the division between the tarsus and metatarsus, and defining the line for Hey's amputation of the foot.

"The measurements of the injured member, as compared with those of the opposite foot, were the following: Length of the dislocated extremity from the point of the great toe to the heel, 9½ inches; of the uninjured foot, 10¼ inches. Breadth of the dislocated foot across its widest part at the base of the great toe, 4¼ inches; of the uninjured foot, 3½ inches. The extensor tendons of the injured foot stood out in strong relief, raising the toes; the tendons of the sound foot could be but indistinctly seen.

"These were the principal appearances which presented themselves. The patient at this time had made no effort to walk, for upon the few occasions on which he had tried to use the limb, supported by crutches, he found a total inability to move otherwise than on the heel, in consequence of pain of a burning, lancinating character, being produced on the sole of the foot, whenever he attempted to throw any weight upon the toes, and to place the plantar structures on the stretch.

"Six months afterwards I obtained a second cast of the foot, and again carefully inspected the limb. It had now become more inverted, and the projection in the sole was less evident, having been rounded and partly removed by absorption. The patient walked freely with a stick, bearing his weight on the outer border of the foot, as in a case of talipes varus, but he could not make any effort at progression, or even move, when the foot was placed flat upon the ground, from the same burning pain before referred to, and which he described as resembling the feeling that might be imagined to result from attempting to walk in a very tight boot with a marble under the sole of the foot."

